# Distinctive roles of Abi1 in regulating actin-associated proteins during human smooth muscle cell migration

**DOI:** 10.1038/s41598-020-67781-1

**Published:** 2020-06-30

**Authors:** Ruping Wang, Guoning Liao, Yinna Wang, Dale D. Tang

**Affiliations:** Department of Molecular and Cellular Physiology, Albany Medical College, 47 New Scotland Avenue, MC-8, Albany, NY 12208 USA

**Keywords:** Cell biology, Cytoskeleton, Actin

## Abstract

Smooth muscle cell migration is essential for many diverse biological processes such as pulmonary/cardiovascular development and homeostasis. Abi1 (Abelson interactor 1) is an adapter protein that has been implicated in nonmuscle cell migration. However, the role and mechanism of Abi1 in smooth muscle migration are largely unknown. Here, Abi1 knockdown by shRNA reduced human airway smooth muscle cell migration, which was restored by Abi1 rescue. Abi1 localized at the tip of lamellipodia and its protrusion coordinated with F-actin at the leading cell edge of live cells. In addition, we identified profilin-1 (Pfn-1), a G-actin transporter, as a new partner for Abi1. Abi1 knockdown reduced the recruitment of Pfn-1 to the leading cell edge. Moreover, Abi1 knockdown reduced the localization of the actin-regulatory proteins c-Abl (Abelson tyrosine kinase) and N-WASP (neuronal Wiskott–Aldrich Syndrome Protein) at the cell edge without affecting other migration-related proteins including pVASP (phosphorylated vasodilator stimulated phosphoprotein), cortactin and vinculin. Furthermore, we found that c-Abl and integrin β1 regulated the positioning of Abi1 at the leading edge. Taken together, the results suggest that Abi1 regulates cell migration by affecting Pfn-1 and N-WASP, but not pVASP, cortactin and focal adhesions. Integrin β1 and c-Abl are important for the recruitment of Abi1 to the leading edge.

## Introduction

Smooth muscle cell migration is essential for many biological processes such as pulmonary/cardiovascular development and homeostasis. Aberrant smooth muscle cell migration contributes to many pathological processes including airway/vascular remodeling^1^. In response to guidance cues such as extracellular matrix proteins, cells expand their membrane at the front to form protrusions (also called lamellipodia), which is critical for directed migration. The formation of cell protrusions is largely driven by local actin filament assembly^1,2^. However, the mechanisms that regulate local actin dynamics are not fully understood.

Abi1 (Abelson interactor 1) is an adapter protein that has been implicated in actin cytoskeletal remodeling^3^, intercellular adhesion^4^, cardiovascular development^5^, nonmuscle cell migration^6,7^ and smooth muscle contraction^8^. Abi1 may complex with SOS1/EP58 to regulate Rac activation and metastasis of ovarian cancer cells^9^. Expression and tyrosine phosphorylation of Abi1 promote adhesion, extracellular matrix degradation and invasion of colorectal carcinoma cells^10^. Nevertheless, the role of Abi1 in smooth muscle migration is largely unknown. Abi1 regulates actin cytoskeletal reorganization by modulating nucleation promoting factors such as N-WASP (neuronal Wiskott–Aldrich syndrome protein) and WAVE (WAS family member), which activate Arp2/3-dependent actin filament branching and assembly^3,8,11^. Abi1 may also interact with Diaphanous formins to promote actin polymerization-driven protrusion formation at the leading edge of motile mouse melanoma B16F1 cells^12^.

Actin cytoskeletal reorganization is also regulated by various actin-regulatory proteins such as cortactin, glia maturation factor-γ (GMFγ), profilin-1 (Pfn-1) and vasodilator-stimulated phosphoprotein (VASP). Cortactin and GMFγ are considered WASP/WAVE/Arp2/3-dependent actin-regulatory proteins. Cortactin directly interacts with N-WASP and activates the Arp2/3-dependent actin branching^13^. GMFγ binds to Arp2/3 complex and induces debranching^14–16^. In contrast, Pfn-1 and VASP are considered WASP/WAVE/Arp2/3-independent actin-regulatory proteins. Pfn-1 facilitates actin polymerization by catalyzing the exchange of actin-bound ADP for ATP and by releasing G-actin from thymosin-β4, which promotes unidirectional addition of G-actin to F-actin^17,18^. VASP promotes actin stress fiber formation and elongation by antagonizing the capping protein CapZ, promoting the transfer of G-actin to the barbed end of growing actin filaments and increasing the bundling of filaments. Phosphorylation at Ser-157 promotes VASP localization to the cell edge^19^. The role of Abi1 in regulating these actin-regulatory proteins during cell migration has not been previously investigated.

c-Abl (Abelson tyrosine kinase, Abl) promotes actin filament assembly via a number of intermediates including cortactin, p130CAS and GMFγ^8,16,20^. c-Abl can regulate the activity of Abi1 by recruiting a multiprotein complex and by directly interacting with Abi1^5,8,20^. Interestingly, Abi1 reciprocally inhibits c-Abl activation in glioblastoma cells^21^. Moreover, β-integrins couple with α-integrins to form the transmembrane adhesion receptors that connect the actin cytoskeleton to the extracellular matrix. In addition to force transmission and adhesion-initiated signaling, β-integrins have been implicated in cell migration^1,20,22,23^.

In this study, we identified Pfn-1 as a new partner for Abi1. In addition to N-WASP, Abi1 regulates recruitment of Pfn-1 to the leading cell edge and migration. Moreover, integrin β1 and c-Abl regulate Abi1 recruitment to the cell edge.

## Results

### Abi1 regulates human airway smooth muscle cell migration

**Figure 1:**
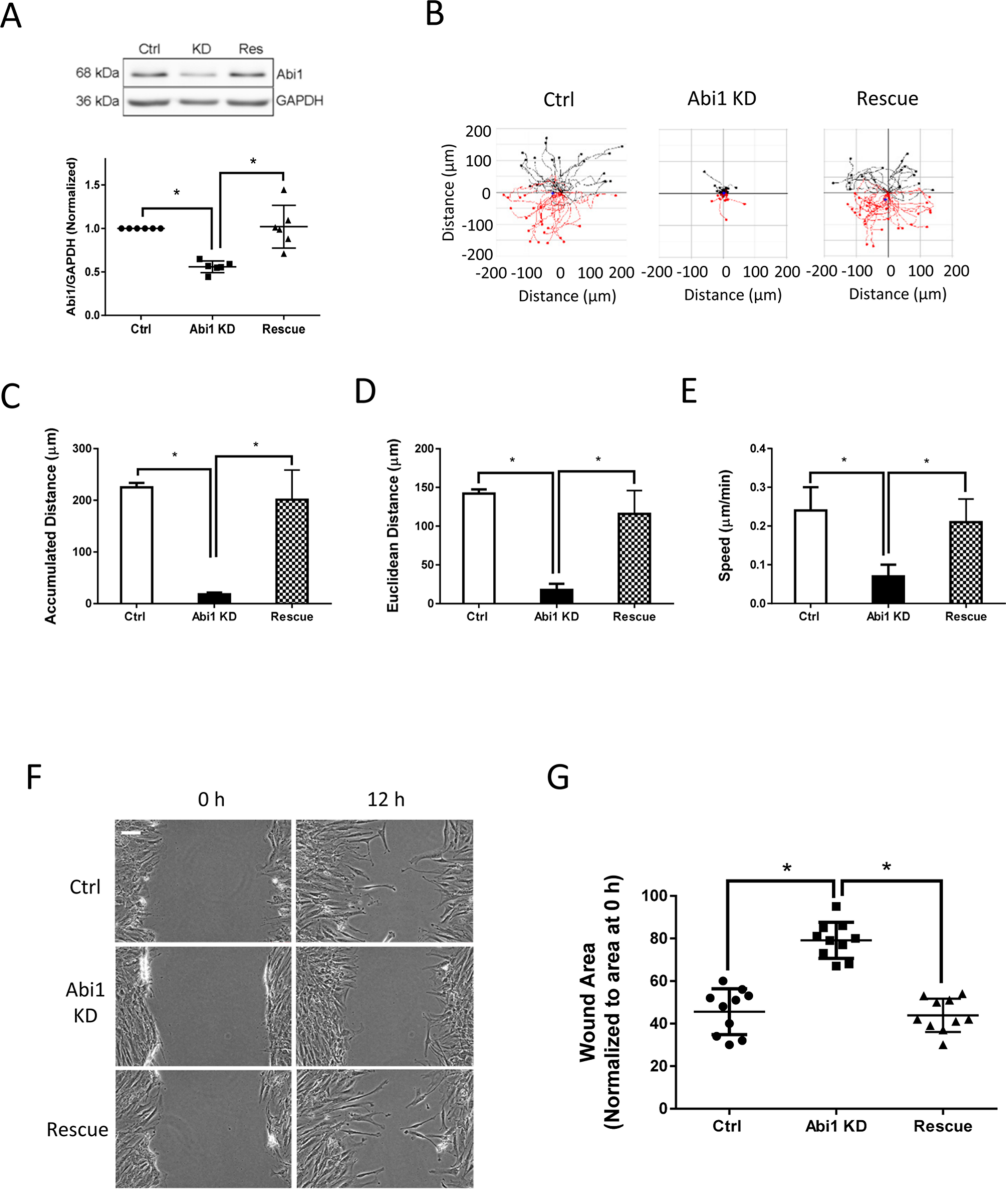
The adapter protein Abi1 promotes cell migration. (**A**) Protein expression of human airway smooth muscle (HASM) cells expressing control (Ctrl) shRNA or Abi1 shRNA, or rescue cells was evaluated by immunoblot analysis. Data are mean values of experiments from 5 batches of cell culture. Error bars indicate SD. (**B**) Migration of HASM cells was tracked using time-lapse microscopy. Images were taken every 10 min for 16 h. Migration plots (35–46 cells from each group) were generated using the NIH ImageJ software. Red tracks indicate downward migration whereas black tracks indicate upward migration. (**C**)–(**E**) Abi1 knockdown (KD) reduced accumulated distance, Euclidean distance and speed of cell migration. Rescue of Abi1 recovered these parameters. (**F**), (**G**) Migration of HASM cells was examined by using the wound healing assay. Scale bar: 100 µm. n = 10 experiments. Error bars indicate SD. *p < 0.05. One ANOVA was used for statistical analysis.

To assess the role of Abi1 in cell migration, we have generated stable Abi1 knockdown (KD) HASM cells by using lentiviruses encoding Abi1 shRNA as previously described^8^. Immunoblot analysis verified Abi1 KD in smooth muscle cells (Fig. 1A). Because Abi1 is involved in cell growth^24^, complete KD of Abi1 impairs cell viability. Thus, we used the experimental condition, in which Abi1 was partially downregulated by approximately 50%, for the following experiments. Moreover, we used lentiviruses encoding Abi1 gene to rescue Abi1 in Abi1 KD cells. Immunoblot analysis confirmed Abi1 rescue in the Abi1 KD cells (Fig. 1A).

We used time-lapse microscopy^16,23^ to assess the role of Abi1 in cell migration. The accumulated distance, Euclidean distance and speed of Abi1 KD cells were reduced as compared to control cells (Fig. 1B–E). Rescue of Abi1 restored smooth muscle cell migration (Fig. 1B–E). In addition, we also used the wound healing assay to determine the role of Abi1. The opening area in Abi1 KD cells after 12 h was greater compared to control cells, which was reversed by Abi1 rescue (Fig. 1F,G). These results suggest that Abi1 plays a role in regulating human smooth muscle cell migration. We also noticed that 50% of Abi1 KD reduced accumulated distance and Euclidean distance by approximately 80%. This implies that the migratory distances of HASM cells are sensitive to Abi1 KD.

### Abi1 complexes with Pfn-1 in smooth muscle cells

**Figure 2:**
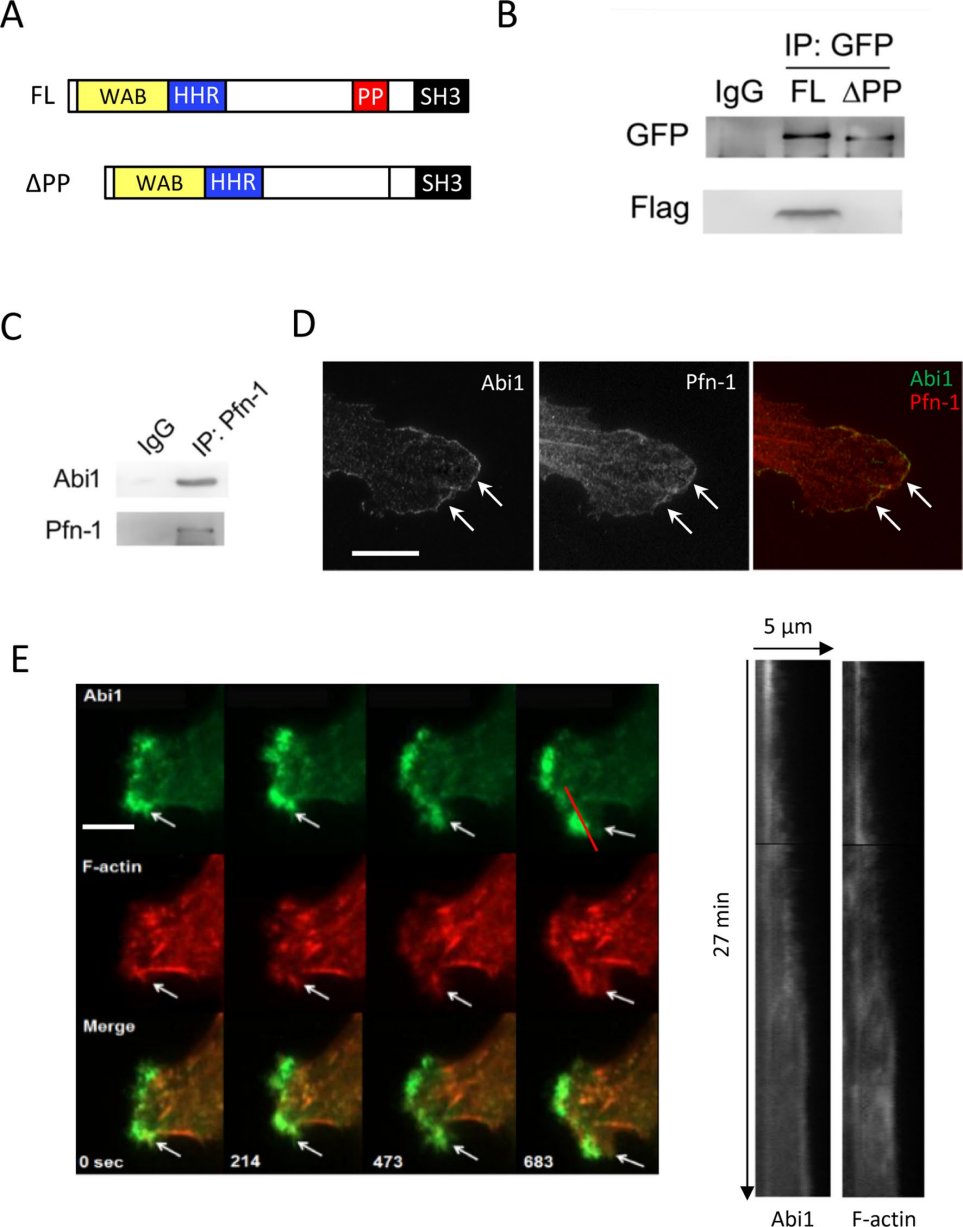
Abi1 complexes with Pfn-1 and they colocalize at the leading cell edge. (**A**) Schematic illustration of Abi1 full-length (FL) and the truncated fragment. Abi1 contains proline-rich (PP) and SH3 domains in its C terminus, and the WAVE binding (WAB) and homeodomain homology region (HHR) in its N terminus. (**B**) FL Abi1, but not Abi1 mutant lacking proline-rich domain (∆PP), complexes with Pfn-1. EYFP-tagged FL Abi1 or ∆PP were cotransfected into cells with Flag-Pfn-1. Cell extracts were immunoprecipitated with GFP antibody and blotted with GFP antibody and Flag antibody. Blots are representative of three identical experiments. (**C**) Extracts of HASM cells were immunoprecipitated with Pfn-1 antibody and blotted with antibodies against Abi1 and Pfn-1. Blots are representative of three identical experiments. (**D**) Abi1 and Pfn-1 were colocalized at the leading edge. Cells were immunostained for Abi1 and Pfn-1, and examined using a confocal microscope. Scale bar: 10 µm. (**E**) Abi1 and F-actin coordinately spread at the cell edge of live HASM cells. Space and time of labeled Abi1 and F-actin in cells were monitored live using a TIRF microscope. The red line indicates the position where kymographs are assessed. Scale bar: 5 µm.

Abi1 is an adapter protein that has potential to interact with other proteins. Because the actin-regulatory protein Pfn-1 is known to regulate cell migration^1,2^, we hypothesized that Abi1 may complex with Pfn-1 in smooth muscle cells. We cotransfected cells with constructs encoding EYFP-tagged full length Abi1 and constructs for Flag-Pfn-1. We used a cross-reactive GFP antibody to detect EYFP-Abi1 and Flag antibody for Flag-Pfn-1 detection. We found that Pfn-1 was in Abi1 immunoprecipitates (Fig. 2A,B). Because Pfn-1 can interact with proline-rich domain of cortactin^25^ and Abi1 possesses a proline-rich domain near its C-terminus^5^, we also assessed whether deletion of Abi1 proline-rich domain affects this protein–protein association. The Abi1 mutant lacking the proline-rich domain lost its complexing with Pfn-1 (Fig. 2A,B). Furthermore, we used coimmunoprecipitation analysis to assess the interaction of endogenous Abi1 with endogenous Pfn-1. Abi1 was found in Pfn-1 immunoprecipitates (Fig. 2C).

### Abi1 and F-actin coordinately protrude at the cell edge of motile smooth muscle cells

Next, we evaluated the cellular distribution of Abi1 with focus on lamellipodia by immunofluorescence microscopy. Abi1 was localized at the tip of lamellipodia of HASM cells (Fig. 2D), which is supported by a previous study by others^7^. In addition, Pfn-1 colocalized with Abi1 at the leading cell edge (Fig. 2D).

Abi1 has been implicated in promoting actin polymerization in vitro^3^. To assess the role of Abi1 in actin dynamics in live cells, we used TIRF (total internal reflection fluorescence) microscopy to monitor space and time of EGFP-labeled Abi1 and RFP-tagged lifeact near surface regions (< 200 nm). Actin dynamics in the near surface region is critical for cell migration^23,26^. We found that Abi1 and F-actin coordinately protruded at the leading cell edge of live cells (Fig. 2E). The results suggest that Abi1 may coordinate F-actin assembly during lamellipodial formation of smooth muscle cells.

### Abi1 affects localization of Pfn-1, but not p-VASP (Ser-157) at leading cell edge

**Figure 3:**
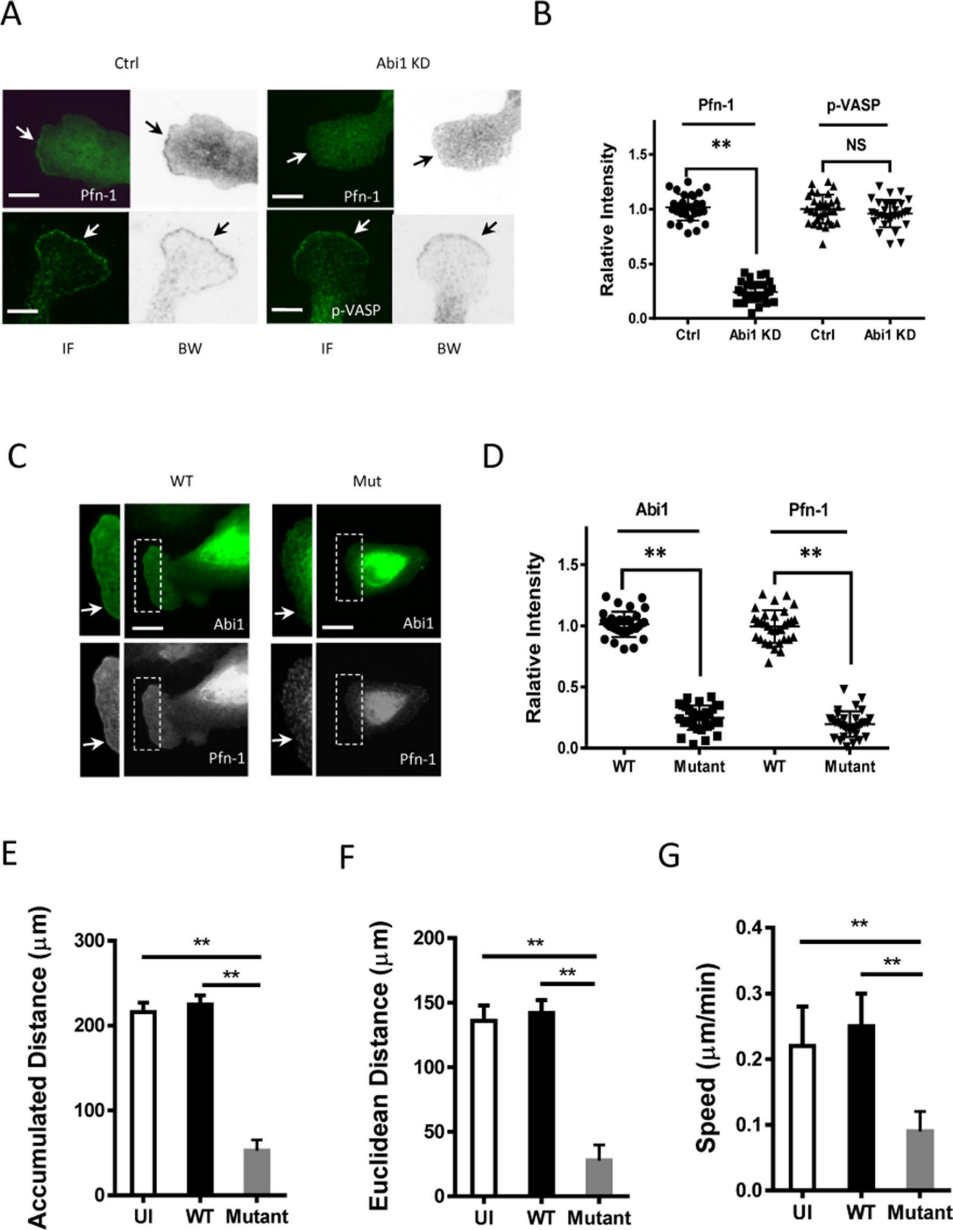
Proline-rich domain of Abi1 is important for leading edge localization of Pfn-1 and cell migration. (**A**), (**B**) Abi1 KD attenuated the cellular localization of Pfn-1, but not pVASP (Ser-157). Immunofluorescent (IF) and black white (BW) images of each cell are shown. BW images of each cell clearly show location of the leading edge. Arrows point to the leading cell edge. Data are mean values from at least 30 cells. Error bars represent SD. Scale bar: 10 µm. (**C**), (**D**) WT Abi1, but not Abi1∆PP mutant, localized at the cell periphery. Also, the expression of the Abi1 mutant reduced the positioning of Pfn-1 at the cell edge. Note: WT and mutant Abi1 also localize in the nuclear/perinuclear region. Data are mean values from at least 32 cells for each group. Error bars represent SD. Scale bar: 20 µm. Arrows point to the leading edge. (**E**)**–**(**G**) WT or Abi1∆PP mutant were re-expressed in Abi1 KD cells followed by assessment of migration. Uninfected (UI) cells were also used as a control. Abi1∆PP mutant inhibited accumulated distance, Euclidean distance and speed of cells (at least 38 cells for each group). Error bars indicate SD. **p < 0.01; NS, not significant. The *t* test was used for statistical analysis.

Pfn-1 and VASP regulate actin polymerization and cell migration by N-WASP-independent pathways. Immunostaining showed that both Pfn-1 and pVASP (Ser-157) (indication of VASP activation)^19^ were positioned at the leading cell edge of smooth muscle cells (Fig. 3A). Next, we evaluated the effects of Abi1 KD on the spatial localization of these two proteins. Abi1 KD inhibited the distribution of Pfn-1, but not p-VASP at the leading edge (Fig. 3A,B). These results suggest that Abi1 regulates the recruitment of Pfn-1, but not activated VASP to the cell edge.

### Proline-rich domain of Abi1 is important for cell migration

Because Abi1∆PP mutant did not bind to Pfn-1 (Fig. 2B), we evaluated whether Abi1∆PP mutant affects the distribution of Pfn-1 in cells. We found that wild type (WT) Abi1, but not Abi1∆PP mutant, localized at the tip of lamellipodia (Fig. 3C). Furthermore, the expression of Abi1∆PP mutant attenuated the distribution of Pfn-1 at the cell edge (Fig. 3C,D). Next, we determined the effects of Abi1∆PP mutant on cell migration by using time-lapse microscopy. Abi1∆PP mutant inhibited accumulated distance, Euclidean distance and speed of cell migration (Fig. 3E–G).

### Abi1 differentially affects localization of c-Abl, N-WASP, cortactin and vinculin in cells

**Figure 4:**
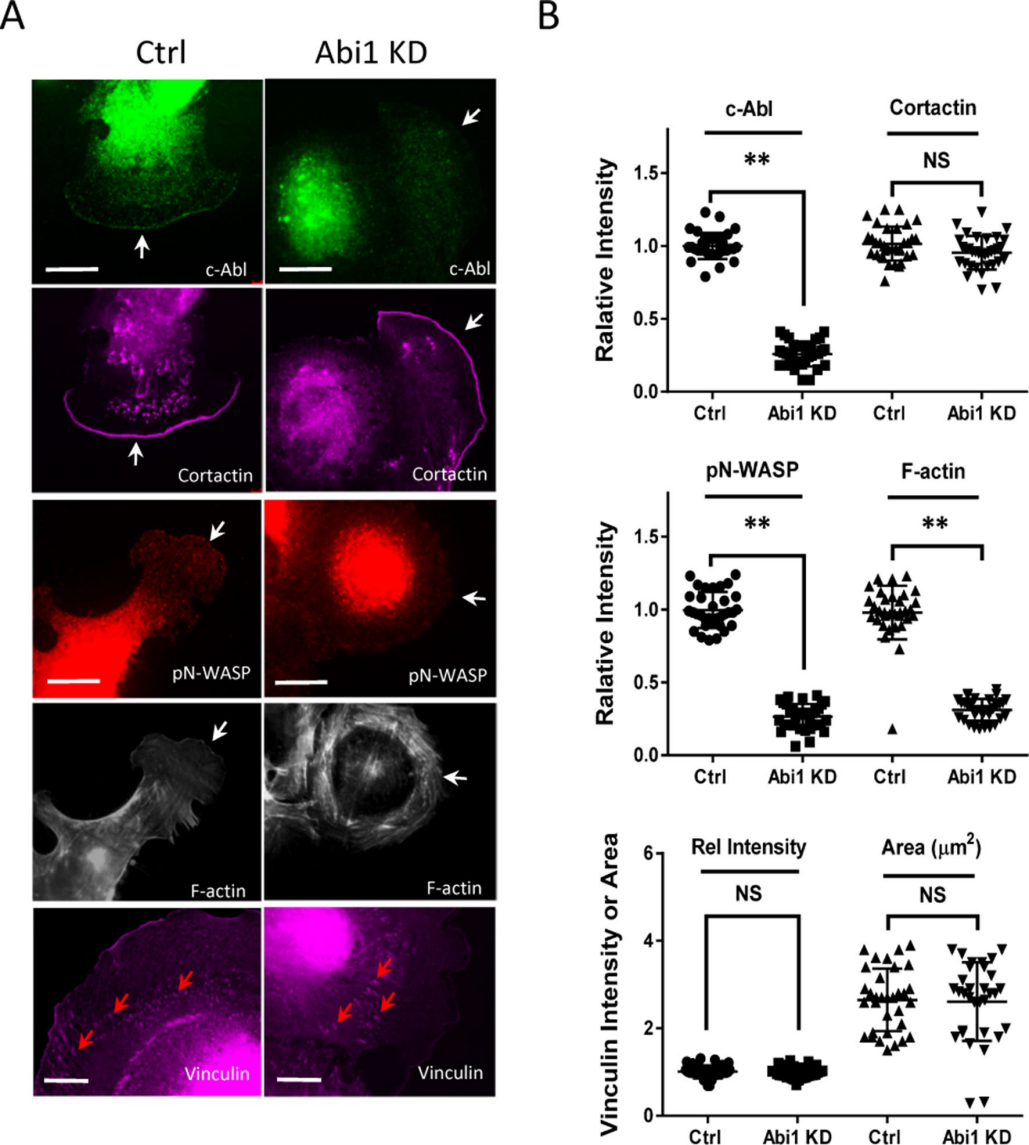
Differential role of Abi1 in spatial localization of migration-associated proteins. (**A**) Abi1 KD attenuated localization of c-Abl, pN-WASP and F-actin at the leading edge without affecting cortactin positioning. In addition, Abi1 KD did not affect vinculin relative intensity and area. Scale bar, 20 µm. White arrows point to the leading edges. Red arrows point to focal adhesions. (**B**) Data are mean values of experiments from at least 32 cells for each group. Error bars indicate SD. **p < 0.01. NS, not significant. The *t* test was used for statistical analysis.

Because c-Abl, N-WASP, cortactin and vinculin are important for the regulation of cell migration, we used immunofluorescent microscopy to determine whether Abi1 regulates distribution of these proteins. Abi1 KD reduced the localization of c-Abl and pN-WASP (Y256) (indication of N-WASP activation)^16^ at the leading cell edge (Fig. 4A,B). Furthermore, Abi1 KD diminished F-actin distribution at the tip of protrusion (Fig. 4A,B). However, cortactin localization at the leading edge was not affected by Abi1 KD (Fig. 4A,B). Moreover, Abi1 KD did not affect distribution of vinculin, a focal adhesion marker (Fig. 4A,B).

### c-Abl tyrosine kinase modulates localization of Abi1 and Pfn-1 at the tip of protrusion

**Figure 5:**
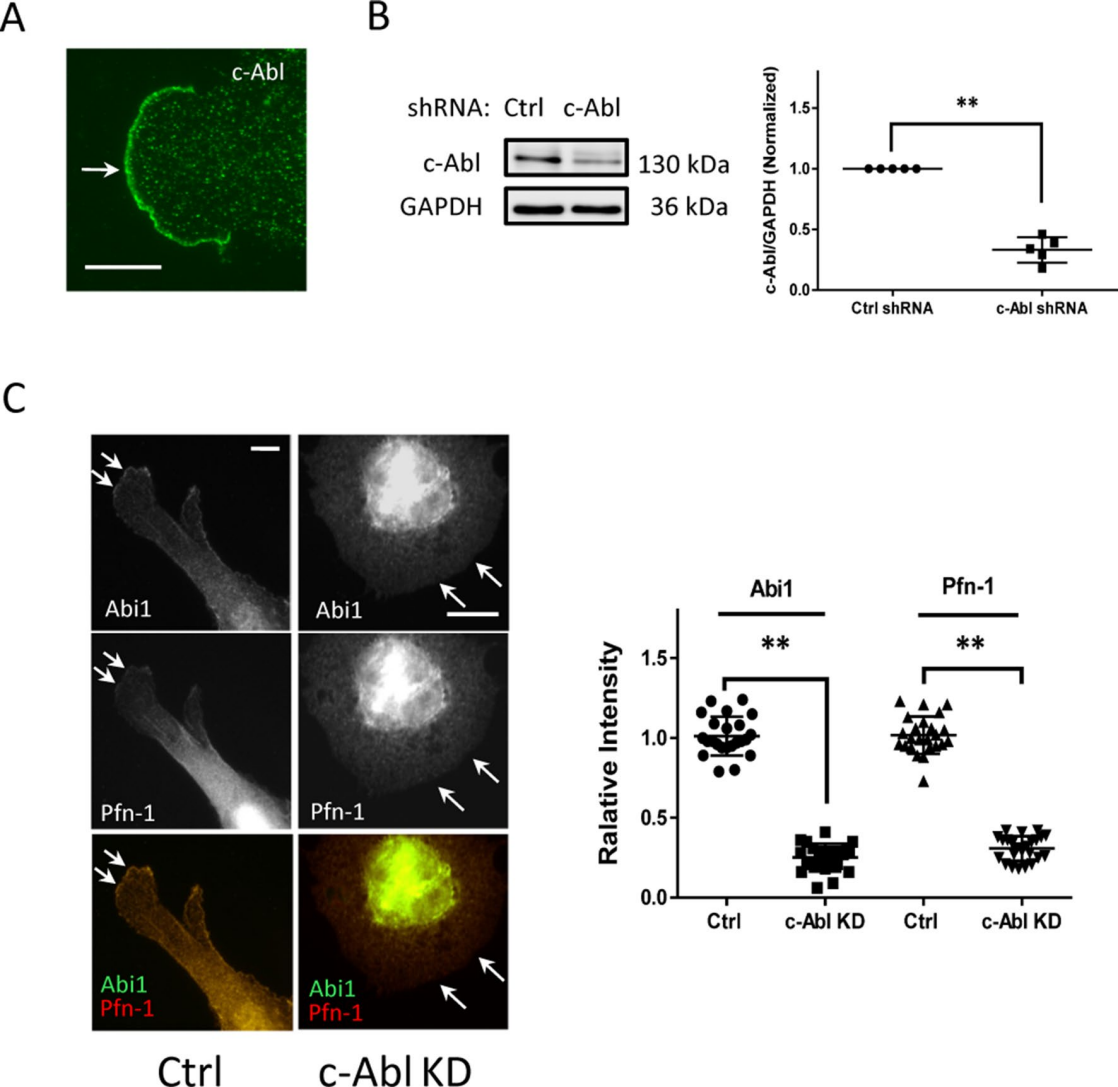
c-Abl regulates the recruitment of Abi1 and Pfn-1 to the leading edge. (**A**) c-Abl is localized at the tip of lamellipodia. Scale bar, 10 µm. (**B**) Immunoblot analysis of stable c-Abl knockdown cells and control cells. Data are mean values of experiments from five batches of cell culture. Error bars indicate SD. (**C**) KD of c-Abl reduced the localization of Abi1 and Pfn-1 at the leading edge. Scale bar, 10 µm. Data are mean values of experiments from at least 30 cells for each group. Error bars indicate SD. **p < 0.01. Arrows point to the leading edge. The *t* test was used for statistical analysis.

c-Abl tyrosine kinase has a role in controlling cell migration^23,27^. We found that c-Abl was concentrated at the leading cell border of motile cells (Fig. 5A), which is supported by previous studies^23^. Thus, we evaluated whether c-Abl regulates the recruitment of Abi1 and Pfn-1. KD of c-Abl reduced the recruitment of Abi1 and Pfn-1 to the leading edge of motile cells (Fig. 5B,C).

### Integrin β1 regulates localization of Abi1 and Pfn-1 at the leading edge

**Figure 6:**
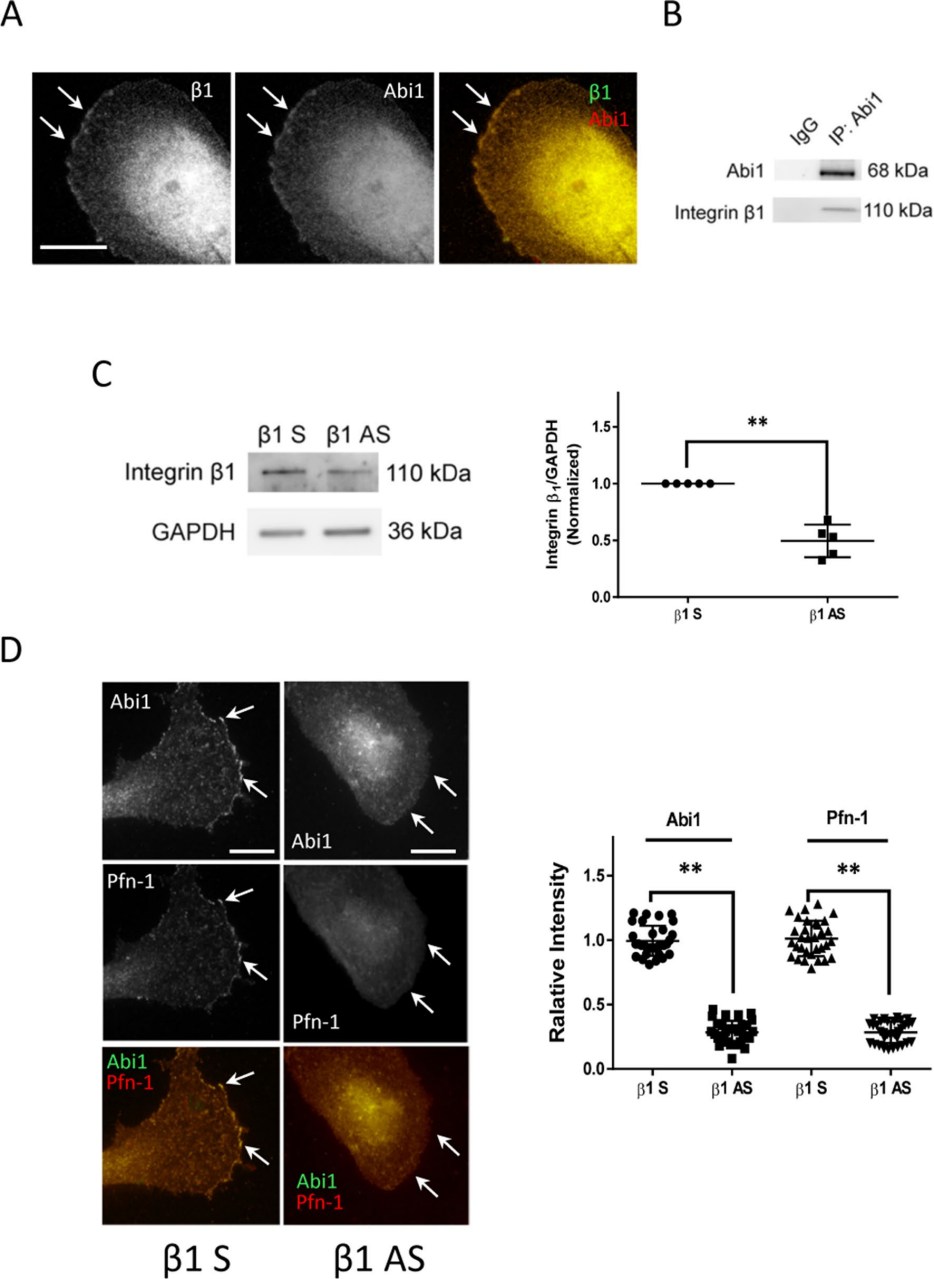
Integrin β1 controls the positioning of Abi1 and Pfn-1 at tip of protrusion. (**A**) Integrin β1 and Abi1 are colocalized in the leading cell edge. Scale bar, 10 µm. (**B**) Coimmunoprecipitation analysis show that Abi1 complexes with integrin β1 in smooth muscle cells. Blots are representative of three identical experiments. (**C**) Immunoblot analysis of HASM cells treated with β_1_ sense (S) or antisense (AS) oligodeoxynucleotides. Data are mean values of experiments from 5 batches of cell culture. Error bars indicate SD. (**D**) KD of integrin β1 reduced the localization of Abi1 and Pfn-1 in the leading edge. Scale bar, 10 µm. Data are mean values of experiments from at least 32 cells for each group. Error bars indicate SD. **p < 0.01. Arrows point to the leading edge. The *t* test was used for statistical analysis.

Integrin β1 is highly expressed in smooth muscle cells and has been implicated in cell migration^1,20,22,23^. We observed that integrin β1 was colocalized with Abi1 at the leading cell edge (Fig. 6A). Furthermore, integrin β1 was found in Abi1 immunoprecipitates of smooth muscle cell extracts (Fig. 6B). Therefore, we evaluated the role of integrin β1 in Abi1 and Pfn-1 distribution. KD of integrin β1 attenuated the localization of both Abi1 and Pfn-1 at the leading edge (Fig. 6C,D).

## Discussion

Membrane protrusion of motile cells is driven by actin polymerization that is controlled by a variety of actin-regulatory proteins including N-WASP, Arp2/3, c-Abl, cortactin, Pfn-1 and VASP. In this study, we found that the adapter protein Abi1 localized to the leading cell edge. Moreover, Abi1 and F-actin coordinately protruded at the leading edge of live cells (Fig. 2E). Knockdown of Abi1 attenuated F-actin assembly at the leading edge (Fig. 4). The results suggest that Abi1 is an important molecule that facilitates actin polymerization-driven lamellipodial formation of motile smooth muscle cells.

Although Abi1 has been implicated in regulating actin dynamics in biochemical and cellular studies, the knowledge regarding the role of Abi1 in smooth muscle cell migration is limited. Here, we discovered that Abi1 plays a positive role in regulating human smooth muscle cell migration, which is supported by previous studies on cancer cell migration^6,7^.

Abi1 is able to complex with Pfn-1, which is mediated by the proline-rich domain of Abi1 (Fig. 2). The N-terminal and C-terminal helices of Pfn-1 form a binding groove for poly-l-proline^28^. Moreover, Abi1 KD reduced Pfn-1 recruitment to the leading edge and cell movement. The Abi1∆PP mutant did not localize at the tip of lamellipodia (Fig. 3C,D). This is not surprising because Abi1 requires the poly-proline domain to interact with the SH3 domain of c-Abl at the cell front^1,20,29^. The localization of Pfn-1 at leading edge and cell migration were also reduced in cells expressing the Abi1∆PP mutant (Fig. 3). Thus, it is likely that poly-l-proline of Abi1 may interact with the binding groove of Pfn-1, which may promote the recruitment of Pfn-1, lamellipodial formation and migration.

Abi1 KD reduced active N-WASP localization to the tip of protrusion (Fig. 4); the result is consistent with biochemical studies, in which Abi1 promotes N-WASP mediated actin polymerization^3,8^. The SH3 domain of Abi1 is sufficient to bind the proline-rich region of N-WASP^3^. Therefore, Abi1 may recruit and activate N-WASP at the leading edge, facilitating actin branching, actin mesh assembly and lamellipodial formation.

However, Abi1 did not have a role in regulating the recruitment of pVASP to the cell edge (Fig. 3A,B). Phosphorylation at Ser-157 by protein kinase A or G facilitates VASP membrane localization^19^, which may promote actin filament assembly and elongation. Moreover, Abi1 KD did not affect cortactin distribution to the tip of lamellipodia (Fig. 4). The recruitment of cortactin to the leading edge may be mediated by Arg^13^. Cortactin may associate with N-WASP and promote N-WASP mediated actin polymerization. Cortactin may also interact with the adapter protein Nck1 and then activate N-WASP^1,13,20^.

Vinculin is one of the major components of focal adhesions, which connect the actin cytoskeleton to the extracellular matrix and are also important for cell migration^1^. Abi1 KD did not affect the dimension of vinculin of motile cells (Fig. 4). The findings suggest that Abi1 does not participate in the assembly of focal adhesions during cell migration. This is not surprising because vinculin-positive focal adhesions localize in lamellipodia of smooth muscle cells (Fig. 4A) whereas Abi1 largely position at the leading cell edge (Fig. 2D). Thus, Abi1 regulates cell movement by affecting membrane protrusion, not by modulating focal adhesion formation.

During cell migration, c-Abl was also positioned at the leading cell edge (Fig. 5A), which is consistent with previous studies^23^. Moreover, c-Abl KD reduced the recruitment of both Abi1 and Pfn-1 to the tip of lamellipodia (Fig. 5B,C). Furthermore, c-Abl recruitment was diminished in Abl1 KD cells (Fig. 4). The results indicate that the recruitment of Abi1 and c-Abl to the tip of lamellipodia is interdependent in motile cells. The interdependent interaction of Abi1 with c-Abl may be mediated by the two forms of intermolecular binding: the SH3 domain of Abi1 with the proline-rich motif of c-Abl, and the poly-proline domain of Abi1 with the SH3 domain of c-Abl^1,20,29^.

Integrin β1 and Abi1 colocalized at the leading cell edge (Fig. 6A), which is similar to other reports that integrin α4 can bind Abi1 and regulates cell spreading, migration and placental/cardiovascular development^5^. Integrin β1 KD reduced the localization of both Abi1 and Pfn-1 at the leading edge of motile cells (Fig. 6C,D). There are two potential mechanisms by which integrin β1 mediate the recruitment of Abi1 and Pfn-1. First, integrin β1 directly interacts with Abi1 in smooth muscle cells. Second, integrin β1 has been shown to regulate c-Abl recruitment to the cell edge^23^. Thus, it is also likely that integrin β1 may recruit c-Abl to the protrusive membrane, which subsequently promotes the redistribution of Abi1 and Pfn-1 to the leading edge.

Smooth muscle migration contributes to airway smooth muscle thickening in allergic asthma. When asthmatic patients are challenged with allergens, the numbers of myofibroblasts (indication of synthetic smooth muscle cells)^30,31^ in the subepithelial area are significantly higher than control as evidenced by structural examination of bronchial biopsy^32^. The luminal border of airway smooth muscle in patients with more severe asthma and airflow obstruction is closer to the epithelium compared to patients with less severe obstruction^33^. In this study, Abi1 plays a role in regulating airway smooth muscle migration, which raises the possibility that Abi1 may participate in the development of airway smooth muscle thickening in asthma. Future studies are required to test the possibility.

**Figure 7:**
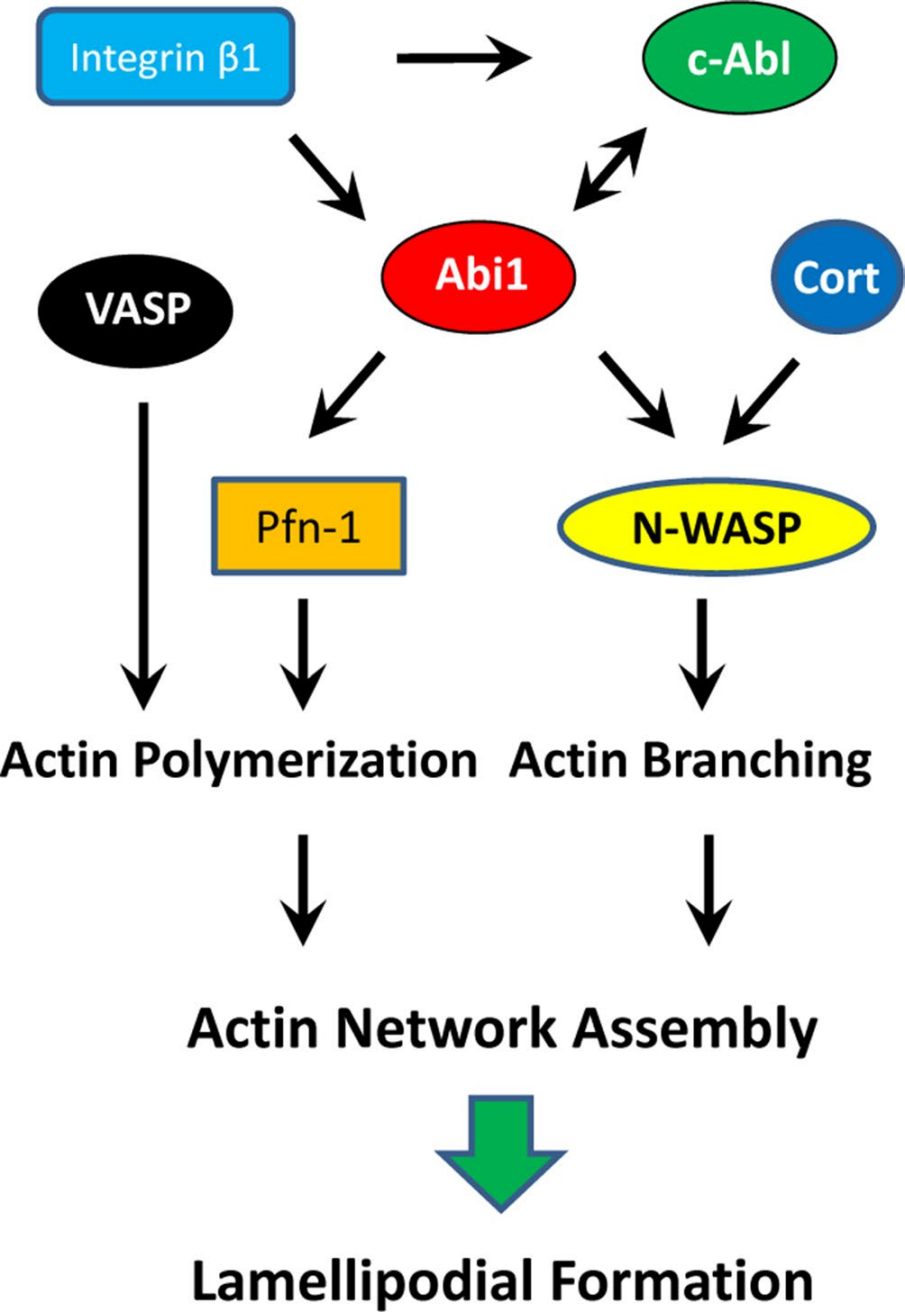
Proposed mechanisms for the regulation of smooth muscle cell migration. In response to extracellular cues (e.g. the ECM), integrin β1 locates to the tip of lamellipodia, which recruits Abi1 to the leading cell edge. Abi1 then recruits Pfn-1, which promotes actin polymerization at barbed end, lamellipodial formation and migration. Abi1 also activates N-WASP, which facilitates actin branching and mesh assembly. Abi1 does not affect the localization and activation of VASP and cortactin (Cort) at the leading edge.

In summary, we identify Pfn-1 as a new partner for Abi1 in human smooth muscle cells. Abi1 localizes to the leading cell edge, which recruits Pfn-1 to the tip of lamellipodia promoting actin polymerization, membrane protrusion and migration. Abi1 also activates N-WASP-mediated F-actin assembly. However, Abi1 does not affect the redistribution of VASP and cortactin (Cort). Abi1 localization is regulated by integrin β1 and c-Abl (Fig. 7).

### Experimental procedures

#### Cell culture

Human airway smooth muscle (HASM) cells were prepared from human bronchi and adjacent tracheas obtained from the International Institute for Advanced Medicine as previously described^8,15,25,34–36^. The Albany Medical College Committee on Research Involving Human Subjects has approved this study. Human lungs were non-transplantable and not identifiable. Informed consented was obtained from all subjects (> 18 years old) for research. All methods were carried out in accordance with the guidelines and regulations of the Albany Medical College Committee on Research Involving Human Subjects. Smooth muscle cells within passage 10 were used for the studies, which is typical for this type of cells^37,38^. α-Actin staining, morphology, proliferation rate and cell size were not significantly different between *passage* 3 and *passage* 12^39^. Primary cells from three donors were used for most experiments. In some cases, duplicate or triplicate experiments from cells of one donor were used for analysis.

#### Time-lapse microscopy

Cells were plated in 6-well culture plates with Ham’s F12 medium supplemented with 10% FBS and cultured in a CO_2_ incubator for 4 h to reach 20–30% of confluence. After serum starvation overnight, culture plates were then placed in a stage incubator filled with 5% CO_2_ at 37 °C. Cell migration was monitored every 10 min for 16 h using a Leica DMI600 microscope system. A 10 ×/dry phase-contrast objective was used for image acquisition. We used the NIH ImageJ software to quantitatively assess accumulated distance, Euclidean distance and speed of cell migration.

#### Wound healing assay

An artificial wound was made in the monolayer of control and Abi1 KD cells by scraping a 10 μl pipette tip across the bottom of the dish. Cells in the medium containing 10% FBS were allowed to migrate for 12 h in a 37 °C incubation chamber with 5% CO_2_. Cell images were taken using a microscope. The remaining open area of the wound was measured using the NIH ImageJ software.

#### Immunoblot analysis and coimmunoprecipitation

Western blotting of cell lysis and coimmunoprecipitation were performed using the experimental procedures as previously described^35,39–42^. Abi1 antibody (1:1,000) was purchased from Sigma (#A5106-200UL, L/N 076M4842V) and validated by using corresponding KD cells. Glyceraldehyde 3-phosphate dehydrogenase (GAPDH) antibody (1:1,000) was acquired from Santa Cruz Biotechnology (#SC-32233, K0315). Flag antibody (1:1,000) was purchased from Sigma (#F3165, L/N SLBH1191V). GFP antibody (1:500) was acquired from Life Technologies (#G-10362, L/N 1696193). Integrin β1 antibody (1:1,000) was purchased from Cell Signaling (#9699S, L/N 1). c-Abl antibody (1:1,000) was purchased from Cell Signaling (#2862S, L/N 15) and was validated by using corresponding KD cells. Antibodies against Flag, GFP and Integrin β1 were validated by examining molecular weight of detected bands. Finally, vendors have provided datasheet to show that antibodies were validated by positive controls. The levels of proteins were quantified by scanning densitometry of immunoblots (Fuji Multi Gauge Software or GE IQTL software). The luminescent signals from all immunoblots were within the linear range.

#### shRNA, antisense oligodeoxynucleotide and plasmids

Stable KD cells were generated using lentiviruses encoding target shRNA as previously described^34,43^. Briefly, lentiviruses encoding Abi1 shRNA (sc-40306-V), control shRNA (sc-108080) and c-Abl shRNA (sc-29843-V) were purchased from Santa Cruz Biotechnology. HASM cells were infected with control shRNA lentiviruses or target shRNA lentiviruses for 12 h followed by 3–4 day culture. We used puromycin to select positive clones expressing shRNAs. The expression levels of Abi1 in these cells were assessed by immunoblot analysis. Abi1 KD cells, c-Abl KD cells and cells expressing control shRNA were stable at least five passages after initial infection. For Abi1 rescue experiment, Abi1 KD cells were treated with lentiviruses encoding RNAi-resistant Abi1 construct. Abi1 rescue in the KD cells was verified by immunoblotting.

For integrin β1 KD, cells were transfected with integrin β1 sense or antisense oligodeoxynucleotides using the Fugene HD transfection reagent kit (Promega) and analyzed 2 days after transfection^23^. The sequence of integrin β1 sense was 5′-GGCCGGGGCATCTGTGAGTG-3′ whereas the sequence of integrin β1 antisense was 5′-CACTCACAGATGCCCCGGCC-3′. The oligodeoxynucleotides were synthesized by Invitrogen.

EYFP tagged FL Abi1 and Abi1 mutant lacking proline-rich domain were gift of Dr. Ann Marie Pendergast of Duke University. In addition, Abi1 was subcloned into pEGFP for live cell imaging. Cell transfection was performed using the Fugene HD transfection reagent kit (Promega) and analyzed 2 days after transfection.

#### Fluorescent microscopy

Cells were plated in dishes containing collagen-coated coverslips and cultured in a CO_2_ incubator for 30 min, followed by fixation and permeabilization^34,43–45^. These cells were immunofluorescently stained using primary antibody followed by appropriate secondary antibody conjugated to Alexa-488, Alexa-555, Alexa-350 or Alexa-647 (Invitrogen, ThermoFisher). For visualization of F-actin, cells were stained with rhodamine-phalloidin. The cellular localization of fluorescently labeled proteins was viewed under a high resolution digital fluorescent microscopy (Leica DMI600) or a Zeiss LSM 880 NLO confocal microscope with Fast Airyscan module (Carl Zeiss Microscopy Jena, Germany). The time of image capturing and intensity gaining were optimally adjusted and kept constant for all experiments to standardize the fluorescence intensity measurements among experiments. The relative intensity is calculated using the following formula: intensity of each cell/average intensity of control cells. For live-cell experiment, cells were transfected with pEGFP encoding Abi1 and mTagRFP-T-lifeact-7 (Addgene) that encodes an F-actin binding peptide tagged with RFP for 48 h. The space and time of labeled proteins were monitored live using a TIRF microscopy (Zeiss).

c-Abl antibody (1:25) was purchased from Cell Signaling (#2862S, L/N 15) and was validated by using corresponding KD cells. Cortactin antibody (1:25) was purchased from Santa Cruz Biotechnology (#sc-55579, L/N E0417) and validated by examining molecular weight of detected bands. Pfn-1 antibody (1:20) was acquired from Sigma (#P8248-200UL, L/N 065K4846) and validated by examining molecular weight of detected bands. pN-WASP (Y256) antibody was purchased from EMD Millipore (#AB1966, L/N 3035272) and validated by examining molecular weight of detected bands. Vinculin antibody was custom prepared by BABCO (Richmond, CA, USA) and validated by determining molecular weight of detected bands and immunostaining. pVASP (S157) antibody was purchased from Cell Signaling (#3111, L/N 1) and validated by determining molecular weight of detected bands.

#### Statistical analysis

All statistical analysis was performed using Prism software (GraphPad Software, San Diego, CA, USA). Differences between pairs of groups were analyzed by Student’s *t* test. A comparison among multiple groups was performed by one-way or two-way ANOVA followed by a post hoc test (Tukey’s multiple comparisons). Values of n refer to the number of experiments used to obtain each value. P < 0.05 was considered to be significant.

## Supplementary information

Supplementary information

## Data Availability

All data generated or analyzed during this study are included in this published article. All research materials are available upon request by qualified investigators.

## Abbreviations

Arp2/3: Actin related protein 2/3
GAPDH: Glyceraldehyde 3-phosphate dehydrogenase
HASM: Human airway smooth muscle
KD: Knockdown
N-WASP: Neuronal Wiskott–Aldrich Syndrome Protein
Pfn-1: Profilin-1
RFP: Red fluorescence protein
RNAi: RNA interference
shRNA: Short hairpin RNA
siRNA: Small interfering RNA
TIRF: Total internal reflection fluorescence
VASP: Vasodilator-stimulated phosphoprotein
WAVE: WAS family member
WT: Wild type
