# Supplementary material for: Distinctive roles of Abi1 in regulating actin-associated proteins during human smooth muscle cell migration
Source: Sci Rep. 2020 Jun 30;10:10667. doi: 10.1038/s41598-020-67781-1 (PMC7326921; doi:10.1038/s41598-020-67781-1)

Supplementary Figure

Distinctive roles of Abi1 in regulating actin-associated proteins during human smooth muscle cell migration

Ruping Wang, Guoning Liao, Yinna Wang and Dale D. Tang*

Department of Molecular and Cellular Physiology

Albany Medical College

47 New Scotland Avenue, MC-8

Albany, New York, U.S.A.

*Correspondence: Dale D. Tang, Department of Molecular and Cellular Physiology, Albany Medical College, 47 New Scotland Avenue, MC-8, Albany, NY 12208
Tel: (518)-262-6416; Fax: (518)-262-8101; E-mail: tangd@mail.amc.edu

Original blots for Figure 1A

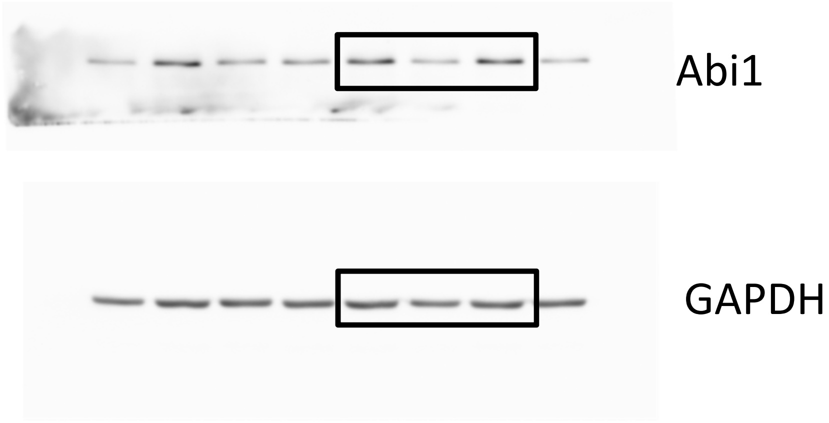

Original blots for Figure 2B

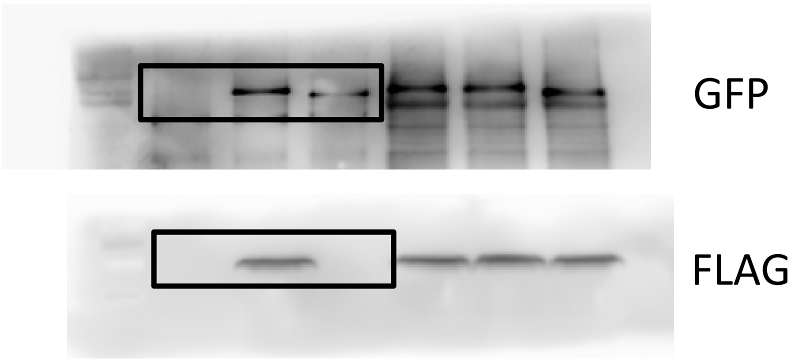

Original blots for Figure 2C

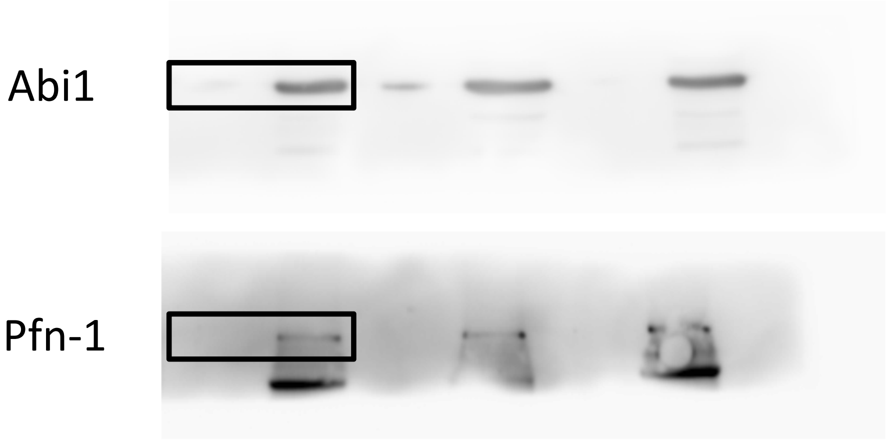

Original blots for Figure 5B.

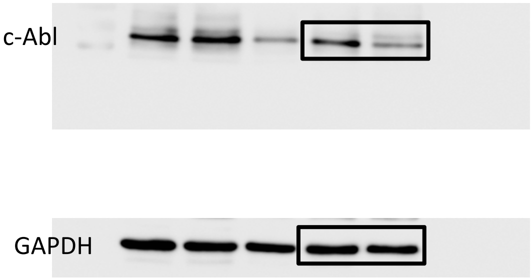

Original blots for Figure 6B

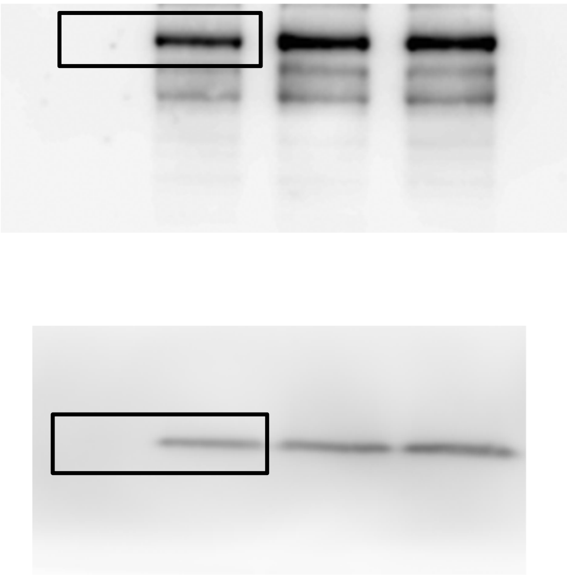

Original blots for Figure 6C

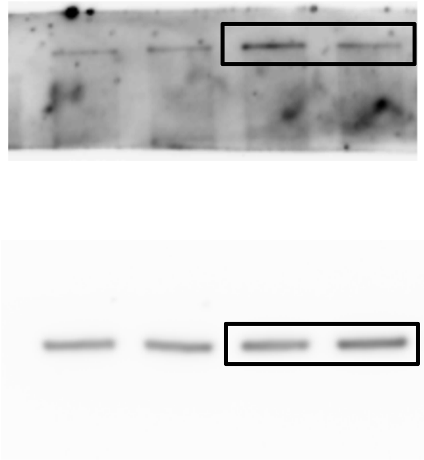

Supplement: Supplementary file 1 — Supplementary information [file 41598_2020_67781_MOESM1_ESM.pdf]
